# Mangrove cyanobacterial diversity as a source of bioactive natural products

**DOI:** 10.1016/j.crmicr.2026.100557

**Published:** 2026-01-23

**Authors:** Arup Ratan Roy, Shayontani Basu, Sergio de los Santos Villalobos, Joydeep Mukherjee

**Affiliations:** a School of Environmental Studies, Jadavpur University, Kolkata, India; b Departamento de Ciencias Agronomicas y Veterinarias, Instituto Technologic de Sonora, Cd. Obregon, Sonora, Mexico

**Keywords:** Cyanobacteria, Mangrove ecosystems, Polyphasic taxonomy, Bioactive natural products

## Abstract

Extensive exploration of lesser-explored habitats undertaken in search of novel and sustainable sources of pharmaceuticals, agrochemicals, renewable energy sources, and dyestuffs has uncovered the capability of cyanobacteria or blue-green algae as producers of these commodities. Mangroves represent one such highly abiotic-stressed and vulnerable biodiversity hotspot found in the intertidal regions of the tropics and sub-tropics that harbor diverse microflora. However, limited data on cyanobacterial taxonomic classification and the study of secondary metabolites rich in novel molecules are currently available, as compared to other prokaryotes. The discovery of useful natural products from cyanobacteria is limited since few strains can be genetically modified, and beneficial compounds are often produced in low or inconsistent amounts. Difficulties with transferring biosynthetic pathways to other hosts, combined with the high cost and ecological challenges of scaling cultivation and advanced screening, require integrated multiomics and simple, scalable workflows for industrial translation. This article attempts to review the reported bioactive secondary metabolites of importance produced by cyanobacteria from mangrove ecosystems across the world and the modern methods that can be utilized for bioactive compound discovery from these cyanobacteria.

## Highlights

• First global review linking polyphasic taxonomy to cyanobacterial metabolites in mangroves.
• Catalogs cyanobacterial strains recognized only by modern morphological and molecular data.
• Reveals high cyanobacterial diversity across mangrove ecosystem, including novel taxa.
• Highlights diverse bioactivities: antimicrobial, anticancer, toxins, bioplastic.
• Bridges taxonomy with biotech applications in drugs, agrochemicals, dyes, biofuels.

## Graphical abstract

**Figure:**
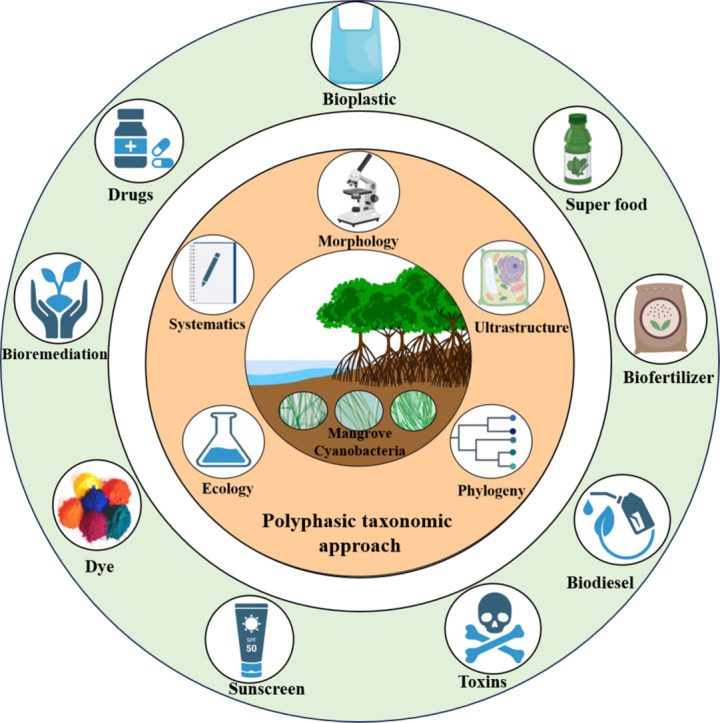

## 1 Introduction

**Fig. 1:**
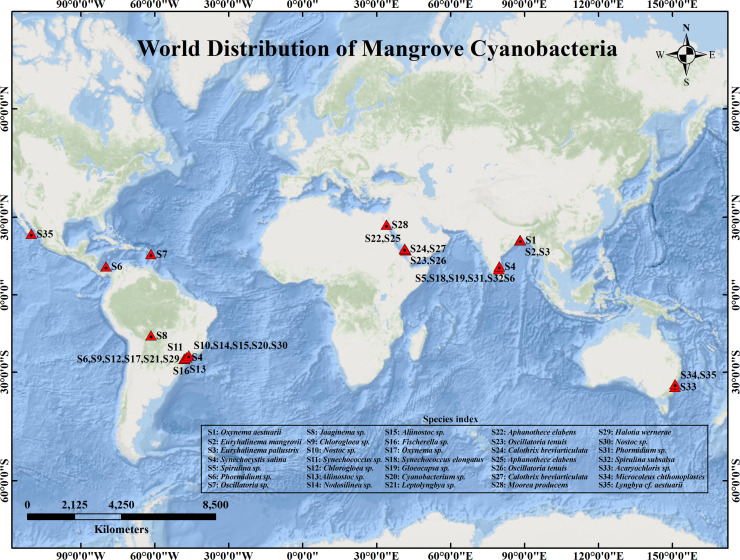
Global distribution of cyanobacterial genera in mangrove habitats. The genera are referred to as S1 to S35 in the map and the respective genera are explained in the key within the map.

**Fig. 2:**
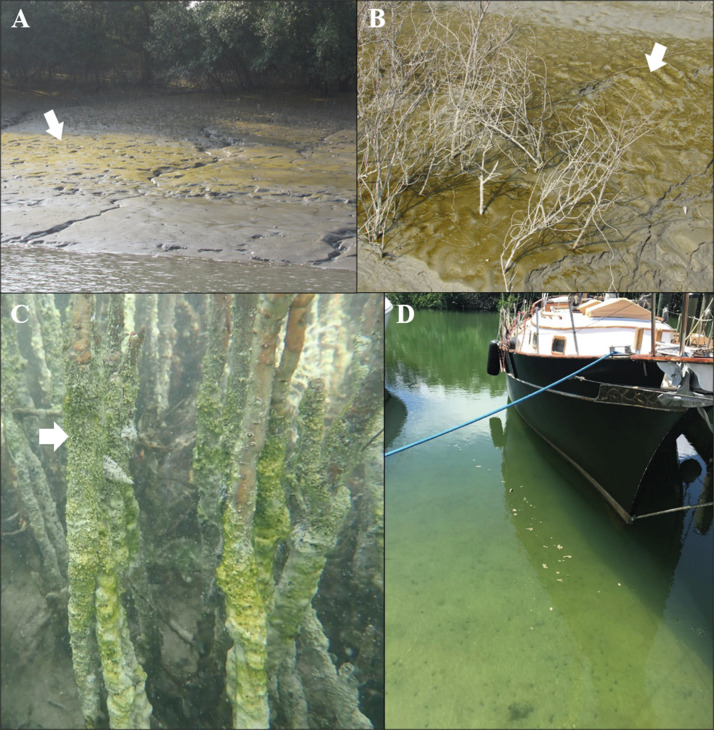
(A and B) Cyanobacterial biofilm formed on the surface of soil sediments of the Sundarbans, India the world’s largest intertidal mangrove forest ecosystem. (C and D) Cyanobacterial biofilm formed on the surface of mangrove roots and soil sediments in the coastal areas of South Florida, USA. The arrow pointed at the cyanobacterial mats. (Image A, B reproduced from (Balu et al., 2020), copyright license No. 5237690888906 and image C, D reproduced from (Berthold et al., 2021), copyright license No. 5237000719750.

Mangroves are unique, highly susceptible, and unevenly distributed ecosystems located along the shorelines of tropical and subtropical regions, hosting a variety of genetically and morphologically diverse organisms. Various microbial communities that are adapted to survive the unstable ecological conditions prevalent in these ecotones reside here by virtue of the abundance of inorganic nutrients and carbon (Giri et al., 2011; Pramanik et al., 2011). These include mainly physical factors such as salinity, temperature, sunlight, availability of gases, tidal fluctuations, and moisture (Rigonato et al., 2013). Cyanobacteria are ancient, oxygenic photosynthetic prokaryotes that played a fundamental role in the evolution of Earth’s atmosphere and continue to be key contributors to global carbon and nitrogen cycles (Sánchez-Baracaldo and Cardona, 2020). Owing to their remarkable ecological diversity across freshwater, marine, terrestrial, mangrove, and intertidal ecosystems, as well as extreme environments such as deserts, hot springs, hypersaline lakes, and polar regions, and their ability to produce bioactive secondary metabolites, they have gained significant attention for biotechnological and environmental applications (Whitton and Potts, 2012). Cyanobacteria derived from mangrove are a notable group of simple filamentous or unicellular, Gram-negative, oxygen-evolving prokaryotes, present globally in virtually all well-illuminated terrestrial and aquatic habitats (Thatoi et al., 2013; Alvarenga et al., 2015) (Fig. 1). Though a primitive group of organisms, they have evolved adaptive strategies to survive environmental extremes such as high temperatures, prolonged UV exposure, hypersaline waters, arid conditions, and freezing temperatures (Alvarenga et al., 2015; Joset et al., 1996). Years of extensive research indicate that cyanobacteria from mangrove ecosystems are abundant sources of both established and novel bioactive compounds like dolastatins, scytonemins, microcystins, and xanthenes, with broad industrial relevance (Pramanik et al., 2011; Thatoi et al., 2013). In the mangrove intertidal zones, at the intersection of land, sea, and air, cyanobacteria endure regular shifts in salinity and tidal immersion, spending roughly half their life cycle exposed to dry, aerial conditions and the other half submerged in brackish water (Zhang et al., 2013) (Fig. 2A and B). All these distinctive features ensure the competitive success of mangrove cyanobacteria by inducing the production of unique bioactive molecules that are quite different from those produced by organisms inhabiting stable ecosystems (Pramanik et al., 2011; Jones et al., 2021). Despite their importance in ecosystem services as well as on a commercial scale for the manufacture of a host of products, relatively few reports on cyanobacterial NPs, especially from the mangrove and marine ecosystems, are currently available (Alvarenga et al., 2015). Detailed reports on bioactive compounds derived from mangrove cyanobacteria are limited to certain organisms only, which are described below. This is probably due to the use of traditional screening of naturally occurring cyanobacterial populations in the mangrove habitat (without the use of polyphasic taxonomy) for desirable compounds, which tend to be disorganized. As a result, expensive retrieval of previously known compounds rather than the discovery of novel molecules is fairly common (Bull et al., 1992). The polyphasic characterization of mangrove cyanobacteria increases our understanding of the mangrove microflora composition and, in turn, leads to a better realization of their potential applicability across various industries.

The present study is a systematic review undertaken to collate reported data on the mangrove cyanobacterial secondary metabolites of commercial importance and the justification of the taxonomic position of these cyanobacteria following the modern polyphasic approach.

## 2 Taxonomy of mangrove cyanobacteria using a polyphasic approach

Cyanobacteria are among the oldest and most adaptable life forms, thriving in extreme places and in mangrove forests, encompassing various families (Abed et al., 2009). Extended evolution has produced great phylogenetic and metabolic variety in cyanobacteria, which produce unique biomolecules that are valuable for biotechnology (Dahms et al., 2006; Bhadury and Wright, 2004). Study of extremophilic cyanobacteria to understand their taxonomy, physiology and metabolic strategies for surviving harsh conditions is gradually gaining importance. Polyphasic taxonomy, enriched with ecological and biogeographic information, has yielded novel cyanobacterial genera and species, which is of paramount importance for discovering new enzymes, proteins, biopolymers, and potential drugs (Zammit et al., 2012; Zammit, 2018; Zammit et al., 2023). For measuring species-level alpha diversity in mangroves, researchers use a polyphasic approach that combines 16S rRNA and 16S–23S ITS sequence analyses with morphology, ultrastructure, and ecological information (Iteman et al., 2000; Nübel et al., 1997; Boyer et al., 2001; Casamatta et al., 2006; Berthold et al., 2021; Komárek, 2016;Komárek, 2018). Since cyanobacteria are morphologically plastic, this combined approach is essential to separate cryptic, morphological, or ecological taxa. Modern phylogeny has been enhanced by phylogenomics, which utilizes large-scale genetic data for exploring evolutionary relationships among cyanobacteria (Zammit et al. 2023). Addition of phylogenomics to polyphasic taxonomy has improved estimation of species limits, uncovered new orders and families, and resolved unclear genus relationships among cyanobacteria (Strunecký et al., 2023). Recent notable examples include the Indian Sundarbans mangrove cyanobacteria *Oxynema aestuarii, Euryhalinema* sp., *Aerofilum fasciculatum, Leptoelongatus littoralis,* and *Almyronema epifania* (Chakraborty et al., 2018, 2019, 2021; Roy et al., 2024) and *Picosynechococcus mangrovensis, Sirenicapillaria*, and *Allocoleopsis* from the Andaman coast mangrove environment of Thailand (Lim Chun Ginn et al., 2025). However, applying polyphasic identification without culturing individual cyanobacterial strains is challenging. Only a few mangrove-derived strains have been described completely by the polyphasic methods (Table 1), revealing significant lacunae in alpha-diversity studies.

**Table 1:** Globally reported novel genus and species described from the mangrove ecosystem based on polyphasic taxonomic identification.

Cyanobacteria strain	Samples collection habitat	Country	References
*Aerofilum fasciculatum*	Soil sediment	India	Chakraborty et al., 2021
*Almyronema epifaneia*	Soil sediment	India	Roy et al., 2024
*Candidatus Planktothricoides niger*	Biofilm on the sediment from the periphyton	Guadeloupe	Guidi-Rontani et al., 2014
*Candidatus Planktothricoides rosea*	Biofilm on the sediment from the periphyton	Guadeloupe	Guidi-Rontani et al., 2014
*Dapisostemon apicaliramis*	Biofilm	Brazil	Hentschke et al., 2016
*Euryhalinema mangrovii*	Soil sediment	India	Chakraborty et al., 2019
*Euryhalinema pallustris*	Soil sediment	India	Chakraborty et al., 2021
*Foliisarcina bertiogensis*	Leaves of *Avicennia schaueriana*	Brazil	Alvarenga et al., 2016
*Halotia wernerae*	Soil sediment	Brazil	Genuário et al., 2015
*Halotia longispora*	Brackish water	Brazil	Genuário et al., 2015
*Halotia branconii*	Soil sediment	Brazil	Genuário et al., 2015
*Kryptousia macronema*	Leaves of *Avicennia schaueriana* and *Merostachys neesii*	Brazil	Alvarenga et al., 2017
*Kryptousia microlepis*	Leaves of *Avicennia schaueriana* and *Merostachys neesii*	Brazil	Alvarenga et al., 2017
*Leptolyngbya indica*	Soil sediment	India	Debnath et al., 2017D
*Leptoelongatus litoralis*	Soil sediment	India	Chakraborty et al., 2019
*Leptochromothrix engenei*	Biofilm from sand sediment	USA	Berthold et al., 2021
*Leptolyngbya* sp.	Soil sediment, Marine water	Brazil	Silva et al., 2014
*Nodosilinea* sp.	Soil sediment, Marine water	Brazil	Silva et al., 2014
*Oxynema* sp.	Soil sediment, Marine water	Brazil	Silva et al., 2014
*Oxynema aestuarii*	Soil	India	Chakraborty et al., 2018
*Okeania hirsuta*	Marine water	Panama	Engene et al., 2013
*Ophiophycus aerugineus*	Biofilm from sand sediment	USA	Berthold et al., 2021
*Phyllonema aviceniicola*	Leaves of *Avicennia schaueriana*	Brazil	Alvarenga et al., 2016
*Streptostemon lutescens*	Biofilm	Brazil	Hentschke et al., 2016
*Vermifilum ionodolium*	Biofilm from sand sediment	USA	Berthold et al., 2021

## 3 Overview of cyanobacterial biosynthetic pathways and strategies for upscaling metabolite production

**Fig. 3:**
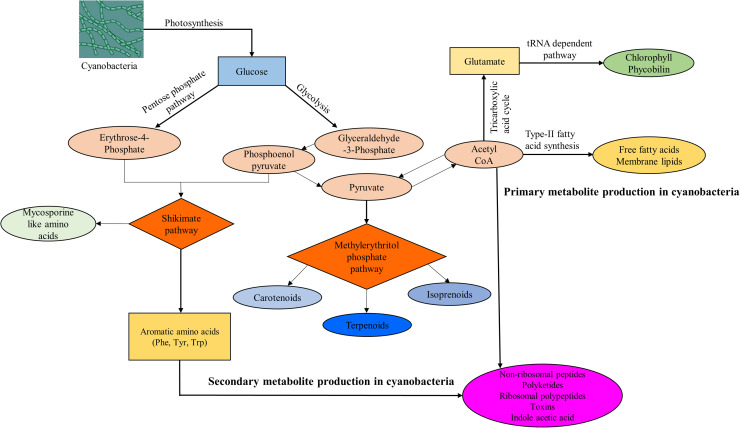
Core pathways for cyanobacterial metabolite biosynthesis, including tRNA-dependent, Type-II fatty acid, shikimate, and MEP pathways, from precursors generated by glycolysis and the pentose phosphate pathway.

Cyanobacterial processes such as photosynthesis and carotenogenesis produce both primary and secondary metabolites. Primary metabolites include antioxidants, structural proteins, and lipids, which support essential functions such as growth, reproduction, and cell division, and can be re‑engineered into products like dyes, biofertilizers, supplements, and bioplastics. Secondary metabolite production varies by species and environment, and commonly serves defensive roles. Examples of cyanobacterial secondary compounds include toxins, phenolics, alkaloids, and antibiotics (Żymańczyk-Duda et al., 2022). The main pathways for primary and secondary metabolites include the transfer ribonucleic acid (tRNA) dependent pathway, Type-II fatty acid synthesis pathway, shikimate pathway, and methylerythritol phosphate (MEP) pathway, with common intermediates and precursors produced by glycolysis and pentose phosphate pathway (Verma et al., 2022; Cao et al., 2023; Nagao et al., 2025). Fig. 3 gives a concise overview of the major biosynthetic pathways for the production of primary and secondary metabolites in cyanobacteria.

Building on this metabolic framework, cyanobacterial natural-product discovery currently involves biosynthetic gene cluster (BGC) identification with compound characterization and scalable production. Approaches including OSMAC (one strain — many compounds) cultivation, native-host genetics, heterologous expression, and in vitro reconstitution are useful for the discovery of silent pathways and expansion of production options. Genomics, metabolomics, and machine-learning tools are used to accelerate dereplication, BGC-metabolite linking, and biotechnological exploitation (Pancrace et al., 2019; Baunach et al., 2024). High‑throughput OSMAC cultivation employing variable nutrients, trace elements, light, CO₂, temperature, desiccation, and chemical elicitors has resulted in conditional production of compounds such as anabaenopeptins, siderophores, and scytonemin (Soule et al., 2007; Demay et al., 2019). In model cyanobacteria such as *Nostoc punctiforme* PCC 73102, transcriptional reporters, forward/reverse mutagenesis, regulator overexpression, and shuttle plasmids may be used to enable BGC activation and mechanistic dissection (Dehm et al., 2019; Baunach et al., 2024; Krumbholz et al., 2022). Since many cyanobacteria yield low metabolite titers or are unfit for manipulation, heterologous routes in engineered *E. coli*, yeast, *Streptomyces*, or cyanobacterial hosts such as *Anabaena* sp. 7120 are used for complementary production and combinatorial chemistry options, though host choice remains empirical (Jones et al., 2012; Dhakal et al., 2021; Baunach et al., 2024). A notable example for enzyme engineering in the context of cyanobacteria associated with the mangrove environment is the development of transgenic *Chlamydomonas reinhardtii* by introducing the cyanobacterial cyanase (CYN) gene from *Synechococcus* elongates (a cyanobacteria with widespread occurrence in mangrove environments) resulting in strong gene overexpression. This engineered strain (*TC. reinhardtii‑2*) displayed enhanced growth, resilience, and efficient removal of cyanide, highlighting its potential as an eco-friendly bioremediation model for polluted freshwater environments (Sobieh et al., 2022). In vitro or chemo‑enzymatic “total biosynthesis” complements cell‑based methods for pathway elucidation and library generation when enzyme function and precursor chemistry are known, exemplified by aetokthonotoxin, cyanobacterin, and guanitoxin studies (Sattely et al., 2008; Adak et al., 2022; D’Agostino et al., 2022; Lima et al., 2022). Integrating genome mining, structural biology‑guided enzyme engineering, microfluidics‑compatible miniaturization, and life‑cycle considerations yields a robust framework that maximizes discovery, mechanistic insight, and routes toward sustainable biotechnological exploitation (Baunach et al., 2024).

Over the past decade, genomics, metabolomics, and bioinformatics have converged to upend natural‐product discovery in cyanobacteria (Dittmann et al., 2015; D’Agostino, 2023; Baunach et al., 2024). Automated platforms such as AntiSMASH predict and annotate BGCs, while spectral networks from Global Natural Products Social Molecular Networking (GNPS) and curated libraries like CyanoMetDB, NPAtlas, and LOTUS accelerate dereplication (Van Der Hooft et al., 2020; Jones et al., 2021; Van Santen et al., 2021). Emerging in silico tools, viz., peptidogenomics suites (NRPquest, MetaMiner, DeepRipp), correlation‐driven metabologenomics, and hybrid frameworks like NPLinker facilitate the application of machine learning to score and visualize BGC–metabolite relationships, rapidly expanding the known chemical space of cyanobacteria (Mohimani et al., 2018; Cao et al., 2019; Merwin et al., 2020). In-silico tools have also been proven useful to detect features relevant for genome editing, notably CRISPR-Cas in the mangrove cyanobacterium *Oxynema aestuarii* AP17. Multiple Type I and Type III CRISPR arrays were characterized in the genome of *O. aestuarii* AP17, along with a previously unreported Type I‑G cas operon, illustrating that mangrove cyanobacteria harbor novel CRISPR systems that could be harnessed for editing or anti‑phage strategies (Basu et al., 2023).

Genome‐scale metabolic models (GSMs) now support both systems biology and biotechnological engineering in cyanobacteria (Goodall et al., 2018; Gale et al., 2019). Updated models for *Synechocystis* PCC 6803, PCC 7942 (iJB785 and iJB792), and UTEX 2973 integrated photosynthetic electron transport, light capture, and O₂ evolution, demonstrating 78–98% agreement with experimental fluxes (Joshi et al., 2017; Broddrick et al., 2019; Gale et al., 2019). Comparative models uncovered strain‐specific carbon uptake and Calvin‐cycle flux differences, guiding in silico optimization of 2,3‐butanediol, n‐butanol (OptFlux), and limonene (OptForce) yields (Ranganathan et al., 2010; Rocha et al., 2010). Yet GSM refinement is limited by sparse proteomic, transcriptomic, and regulatory data (Gale et al., 2019). To close these gaps, the CyanoSource platform has been launched, with a standardized knockout library of over 3,400 genes in PCC 6803 using modular cloning and conditional alleles. Such resources will enhance functional annotation, reproducibility, and the scalable exploitation of mangrove cyanobacterial biosynthetic capacity.

## 4 Types of bioactive compounds produced by mangrove cyanobacteria

**Fig. 4:**
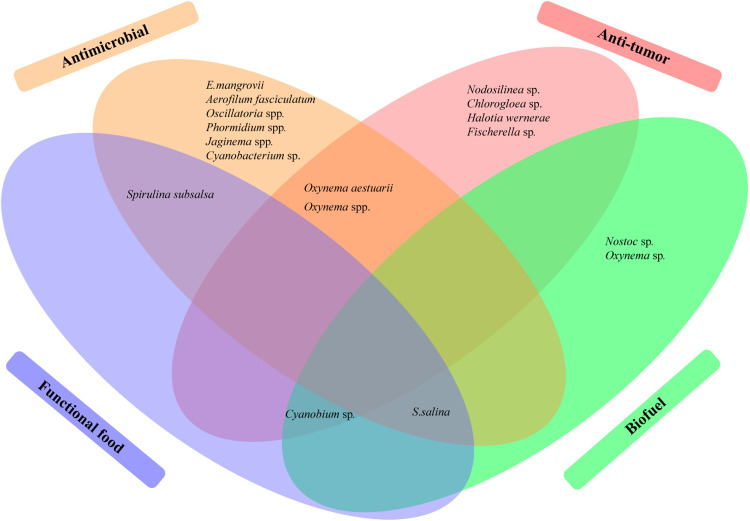
Oval flower diagrams of mangrove cyanobacterial genera with antimicrobial, anti-tumor, biofuel, and functional food activities. The overlaps denote cyanobacteria that have multiple bioactivities.

The taxonomic advances and the biosynthetic strategies outlined in the previous sections establish mangrove cyanobacteria as a valuable resource for natural product discovery. Their diverse lineages, backed by polyphasic taxonomy and phylogenomics, provide a strong basis for linking genetic identity with metabolite production. At the same time, modern approaches for biosynthetic pathway analysis and scaling methods demonstrate how mangrove cyanobacteria can be applied for utilization across various industries. Within this framework, the following section provides a comprehensive overview of the major classes of bioactive compounds reported from mangrove cyanobacteria, emphasizing their ecological functions and commercial relevance in antimicrobial, anti-inflammatory, toxin, photoprotective, pigment, and functional food applications. Table 2 and Fig. 4 display the major categories of NPs from various genera of mangrove cyanobacteria.

**Table 2:** Overview of the bioactive compounds and beneficial primary metabolites produced by mangrove cyanobacteria.

Strains	Compound/ Extract and	Taxonomic classification	Reference
*Planktothrix mougeotii* PMC 1114.19	Godavarin K (Anti-helminthic)	16S rRNA and morphological	Wang et al., 2023
*Oxynema aestuarii, Euryhalinema mangrovii, Euryhalinema pallustris, Aerofilum fasciculatum, Leptoelongatus littoralis*	Methanol extracts, Antimicrobial (Inhibition of *S. aureus, E. coli, B. subtilis, P. aeruginosa*)	Polyphasic taxonomy	Pramanik et al., 2011; Chakraborty et al., 2018, 2019, 2021
*Synechocystis salina, Oscillatoria salina, Spirulina subsalsa, Phormidium fragile, Oscillatoria cortiana, Oscillatoria willei, Phormidium tenue*	Methanol / methanol:chloroform:water extracts, Antifungal (Inhibition of fungi *F. solani, A. flavus*)	Morphological (Desikachary)	Sakthivel and Kathiresan, 2013
*Synechococcus* sp.*, Gloeocapsa* sp.	Aqueous extracts Antimicrobial (inhibit *P. vulgaris* and *P. aeruginosa*)	16S rDNA	Anburaj et al., 2020
*Oscillatoria* sp., *Jaaginema* sp.	Methanol:acetonitrile:water extracts, Antimicrobial Inhibit *E. coli* and *P. atlantica*	16S rRNA	Duperron et al., 2019
*Cyanobium, Chlorogloea, Cyanobacterium, Oxynema, Synechococcus, Leptolyngbya, Nodosilinea, Nostoc*	Ethyl acetate, chloroform, methanol extracts, Antimicrobial (inhibit *B. cereus, S. typhimurium* and *Candida* sp*.*)	16S rRNA	Silva et al., 2014; Silva-Stenico et al., 2014
*Fischerella* sp., *Aliinostoc* sp.	Methanol extracts (Inhibition of *Staphylococcus* sp. and *Candida albicans*)	16S rRNA	Shishido et al., 2019
*Oxynema* sp. AP17	Ethyl acetate extract (anti-angiogenic)	Polyphasic taxonomy	Basu et al., 2021
*Cyanobium* sp., *Oxynema* sp.	Ethanol extracts (CT-26 colon carcinoma inhibition)	16S rRNA	Silva-Stenico et al., 2014; Genuário et al., 2019
*Nodosilinea* sp., *Chlorogloea* sp., *Cyanobium* sp., *Halotia wernerae, Fischerella* sp.	Methanol extracts (cytotoxic to MOLM-13 leukemia cells)	16S rRNA	Silva-Stenico et al., 2014; Shishido et al., 2019
*Aphanothece elabens, Lyngbya majuscula, Leptolyngbya tenuis, Oscillatoria accuminata, Oscillatoria tenuis, Calothrix breviarticulata, Aliinostoc bakau* sp. nov.	Microcystins, saxitoxin	Morphology, Polyphasic taxonomy for *A. bakau*	Mohamed & Al-Shehri, 2015; Merican et al., 2023
*Moorena producens*	Apratoxins, aeruginosin	16S rRNA	Thornburg et al., 2013
*Oscillatoria sp. Gwada, Candidatus Planktothricoides niger, Candidatus Planktothricoides rosea, Cyanobium sp. CENA166, Synechococcus CENA170, Nostoc (Aliinostoc) sp. CENA175, Cyanobium sp. CENA153, CENA154, CENA185, Fischerella sp. CENA161*	Cyanotoxins	16S rRNA	Guidi-Rontani et al., 2014; Genuário et al., 2019; Silva-Stenico et al., 2012; Shishido et al., 2019
*Lyngbya* cf. *aestuarii, Microcoleus chthonoplaste*s	Scytonemin, MAAs	Morphology	Karsten et al., 1998; Singh et al., 2020
*Oxynema aestuarii*	Phycocyanin	Polyphasic taxonomy	Hazra & Saha Kesh, 2017;
*Acaryochloris* sp.	Chlorophyll-d	16S rRNA	Larkum et al., 2012
*Spirulina subsalsa*	Carbohydrates, proteins, lipids, vitamins, minerals	Morphological	Sakthivel & Kathiresan, 2013
*Synechocystis* sp.	Fatty acids (linoleic acid, α-linolenic acid)		Matsudo et al., 2020
*Phormidium* sp.*, Microcoleus chthonoplastes, Phormidium, Microcoleus, Oscillatoria*	Indole acetic acid, EPS, secondary metabolite mixture	16S rRNA; morphological	Boopathi et al., 2013; Sakthivel & Kathiresan, 2015; Bashan et al., 2000
*Synechocystis* sp.	Polyhydroxyalkanoates	16S rRNA	Gracioso et al., 2021

### 4.1 Antimicrobial compounds from cyanobacteria

**Fig. 5:**
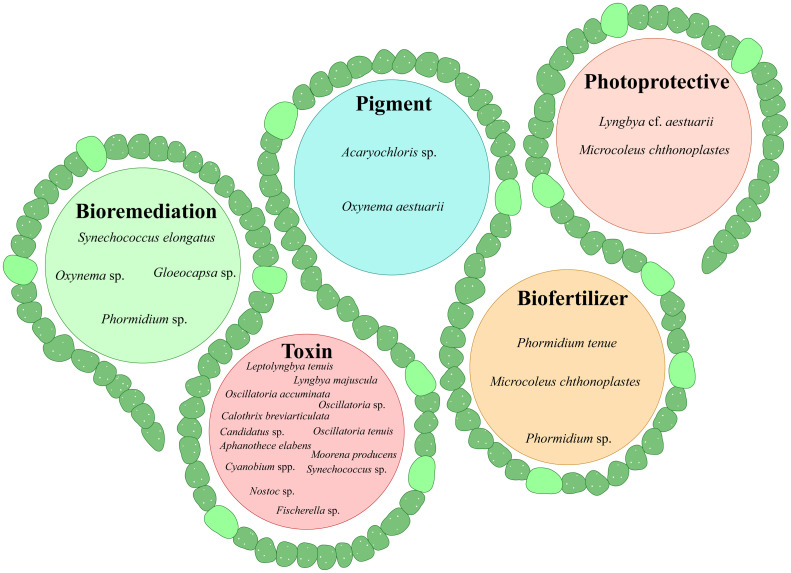
Diagram of mangrove cyanobacterial genera producing pigments, biofertilizers, photoprotective compounds, and toxins, including bioremediating species.

Cyanobacteria have been shown to produce potent antimicrobial compounds, which are gradually gaining importance as many traditional antibiotics fail due to rising resistance (O’Neill, 2016). The majority of the cyanobacterial NPs showing bioactivity are products of polyketide synthase (PKS), nonribosomal peptide synthetase (NRPS), hybrid (NRPS‐PKS), or post-translationally modified ribosomes (RiPPs) (Alsenani et al., 2020). Unsaturated fatty acids and terpenoids of microalgae and cyanobacteria have chiefly been found to possess potent antimicrobial properties (Carpine and Sieber, 2021; Welker et al., 2012, (Kini et al., 2020). A recent study found that *Planktothrix mougeotii* PMC 1114.19, a filamentous cyanobacterial strain isolated from mangrove biofilms in Mayotte island, produces the anti-helmintic and anti-biofilm triterpenoid godavarin K (Wang et al., 2023). Organic solvent extracts of various mangrove-dwelling cyanobacteria isolated from Indian and Brazilian mangrove environments (further elaborated below) have been demonstrated to show antimicrobial activity and can therefore be considered as potential candidates for drug development (Fig. 5). Research indicates that methanol extracts from newly described cyanobacteria, including *Oxynema aestuarii, Euryhalinema mangrovii, Euryhalinema pallustris, Aerofilum fasciculatum*, and *Leptoelongatus littoralis,* effectively inhibit the growth of *Staphylococcus aureus, Escherichia coli, Bacillus subtilis, Pseudomonas aeruginosa*, and multiple drug-resistant bacterial isolates (Pramanik et al., 2011; Chakraborty et al., 2018, 2019, 2021). Based on the modern taxonomic identification using a polyphasic approach, which includes 16S rRNA phylogeny and 16S-23S ITS secondary structure analysis, *O. aestuarii* belongs to Microcoleaceae, *E. mangrovii* and *L. litoralis* belong to Leptolyngbyaceae, and *A. fasciculatum* belongs to Oculatellaceae and *E. pallustris* to Prochlorotrichaceae families, respectively (Chakraborty et al., 2018, 2019, 2021). Research has demonstrated that methanol and methanol:chloroform: water extracts from cyanobacteria, including *Synechocystis salina, Oscillatoria salina, Spirulina subsalsa, Phormidium fragile, Oscillatoria cortiana, O. willei,* and *Phormidium tenue,* inhibit various human pathogens and fungi such as *Fusarium solani* and *Aspergillus flavus* (Sakthivel and Kathiresan, 2015). All the above-mentioned cyanobacteria were classified using only on morphological features following Desikachary (1959) (Desikachary, 1959). Aqueous extracts of *Synechococcus* sp. and *Gloeocapsa* sp. were shown to hinder the development of *Proteus vulgaris* and *Pseudomonas aeruginosa* (Anburaj et al., 2020b). These cyanobacterial strains were identified based on 16s rDNA identification, but phylogeny and NCBI accession numbers were undisclosed. Methanol:acetonitrile: water extracts of members of the *Oscillatoria* and *Jaaginema* genera have been shown to inhibit pathogenic *E. coli* and environmental *P. atlantica* (Duperron et al., 2019). In this literature, the authors studied ten strains of the Oscillatoriales family and eight strains of the Synechococcales family, including *Jaaginema* sp. Although these strains were identified based on only 16s rRNA sequence analysis, it is essential to review and update the taxonomic positions of these cyanobacteria of potential value using the polyphasic approach, to determine the cultivable strains and choices for strain improvement (Bagchi and Singh, 2019; Baunach et al., 2024).

Extracellular ethyl acetate and chloroform extracts and intracellular methanolic extracts of several members of the genera *Cyanobium, Chlorogloea, Cyanobacterium, Oxynema, Synechococcus, Leptolyngbya, Nodosilinea*, and *Nostoc* were proven to be effective inhibitors of several pathogenic bacteria and yeast such as *Bacillus cereus, Salmonella typhimurium,* and *Candida* sp*.* (Silva et al., 2014; Silva-Stenico et al., 2013).

Methanolic intracellular extracts of various isolates of *Fischerella* sp. and *Aliinostoc* sp. were also reported to inhibit *Staphylococcus* sp. and *Candida albicans* (Shishido et al., 2019). *Fischerella* is reported under the Haplosiphonaceae family according to modern cyanobacteria taxonomic classification (Komárek, 2014). The taxonomic assignment of all the strains was based solely upon a 16S rRNA phylogenetic tree study, which is appropriate for genus-level identification. Precise species-level identification necessitates examining the secondary structures of the 16S-23S ITS regions, which are lacking for these strains.

### 4.2 Antitumor and anticancer activity

Owing to their unique biological profiles, several marine cyanobacterial NPs, such as curacin A, dolastatins 10 and 15, and their chemically synthesized analogs, are currently undergoing studies for further drug development (Tan, 2007). These compounds and their analogs which display anticancer activity can cause the potential destruction of various cancer cells through inducing apoptosis, or alteration of cell signaling activity (primarily involving the proteins belonging to the kinase- C family), and various other modes of cancer cell impairment viz. arrest of the cancer cell cycle, induction of dysfunctions of cancer cell mitochondria and oxidative damage to cancer cells (Qamar et al., 2021). Investigations of metabolites extracted from mangrove cyanobacteria isolated from the Indian Sundarbans and various mangrove forests in Brazil (described below) revealed potent anti-inflammatory and anti-cancer activity. Recent findings, indicate that ethyl acetate extracts of the mangrove-inhabiting cyanobacterium *Oxynema* sp. AP17 exhibits noteworthy anti-angiogenic activity (Basu et al., 2021). Research shows that ethanol extracts from *Cyanobium* sp. and *Oxynema* sp. effectively suppress the growth of CT-26 colon carcinoma cells (Silva-Stenico et al., 2014; Genuário et al., 2019). Methanol extracts of *Nodosilinea* sp., *Chlorogloea* sp., *Cyanobium* sp., *Halotia wernerae*, and *Fischerella* sp. were reported to be cytotoxic to the human leukaemia MOLM-13 cell line (Silva-Stenico et al., 2014; Shishido et al., 2019). However, though *Oxynema* sp. AP17 was identified by the polyphasic approach (Chakraborty et al., 2018); the rest of the strains mentioned in this section were identified on the sole basis of 16S rRNA sequence.

### 4.3 Toxins

Mangrove-dwelling cyanobacterial species such as Aphanothece elabens, Lyngbya majuscula, Leptolyngbya tenuis, Oscillatoria accuminata, Oscillatoria tenuis, Calothrix breviarticulata (Mohamed and Al-Shehri, 2015), Moorena producens (Thornburg et al., 2013), Oscillatoria sp. Gwada, Candidatus Planktothricoides niger, Candidatus Planktothricoides rosea (Guidi-Rontani et al., 2014), Cyanobium sp. CENA166, Synechococcus CENA170, and Nostoc (Aliinostoc) sp. CENA175 (Genuário et al., 2019; Silva-Stenico et al., 2012), Cyanobium sp. CENA153, Cyanobium sp. CENA154, Fischerella sp. CENA161, Cyanobium sp. CENA185 (Shishido et al., 2019) have been known to produce toxins that are lethal to most commercially important aquatic finfish and shellfish (and subsequently to the population consuming them). Toxins are usually produced by mat-like massive cyanobacterial growths known as harmful blooms (or cyano-habs), such as those formed by Oscillatoria sp. Gwada, Candidatus Planktothricoides niger, Candidatus Planktothricoides rosea (Guidi-Rontani et al., 2014). 16S rRNA analysis shows that these organisms belong to the Oscillatoriales family of cyanobacteria. Anthropogenic activity-mediated degradation of the mangroves is believed to be the chief cause of the uncontrolled growth of cyano-hab-forming cyanobacteria (Singh and Babele, 2020). The discharge of industrial effluents containing massive volumes of phosphorus and nitrogen into riverine and estuarine waters and gradually escalating temperature and CO_2_ levels are some of the factors contributing to the worldwide increase of cyano-hb occurrences (Singh and Babele, 2020; Paerl and Otten, 2013; Sandrini et al., 2016). The NPs produced by cyano-habs (cyanotoxins) are known to possess distinct structures and are highly toxic to animals and plants even at trace levels (Pearson et al., 2010).. Phylogenetic analysis using 16S rRNA shows the strain belongs to Moorena producens, which was formerly misspelled as Moorea sp. (Thornburg et al., 2013). Moorena producens is reported under the Oscillatoriacae family of cyanobacteria. Microcystin is a hepatotoxin that is produced by Leptolyngbya tenuis, Oscillatoria tenuis, Aphanothece elabens, Cyanobium, Fischerella, and Calothrix breviarticulata. Alkaloids (such as saxitoxin produced by Lyngbya majuscula, Oscillatoria accuminata, and Leptolyngbya tenuis) act as neurotoxins (Shishido et al., 2019; Mohamed and Al-Shehri, 2015). Merican and colleagues (2023) reported a novel species, Aliinostoc bakau sp. nov., as a producer of microcystins. This cyanobacterium was isolated from a mangrove-associated biofilm in Malaysia and subsequently classified using polyphasic taxonomic methods. Some of these above-described toxins, notably apratoxins, and aeruginosin have potential anti-cancer, and trypsin and thrombin inhibiting activity respectively (Thornburg et al., 2013; Silva-Stenico et al., 2012). The toxin-secreting capability of cyano-habs is a cause of concern to the overall health of the mangrove ecosystems due to the risk of biomagnification of the toxins in higher trophic levels of the food web initiated by primary consumers that feed on toxic cyanobacteria. Thus arises the need to monitor mangrove regions for detecting the presence of cyano- habs and their secreted cyanotoxins in a bid to prevent their undesirable effects (Singh and Babele, 2020). Thorough polyphasic and systematic identification of toxin-producing cyanobacteria, incorporating both molecular markers and morphological traits, is vital for fully assessing the risks they pose to animal and human health when present in drinking water (Wilmotte et al., 2017). This is because different members of the same or different species, as well as various cyanobacterial populations, can be either toxic or nontoxic, with toxic members having variable levels of toxins per cell.

### 4.4 Photoprotective compounds

Cyanobacteria, especially those dwelling in the mangrove habitats (notably *Lyngbya* cf. *aestuarii* and *Microcoleus chthonoplastes*), are exposed daily to intense solar radiation by their latitudinal location in the tropical and subtropical coastal intertidal regions (Karsten et al., 1998; Singh et al., 2020). *Lyngbya* is a member of the Oscillatoriaceae family capable of producing photoprotective compounds. Like other photosynthetic organisms, mangrove cyanobacteria synthesize UV protective molecules such as scytonemin to overcome UV exposure-induced cellular damage (caused chiefly by shortwave UV- B radiations of wavelength ranging from 280 to 350 nm) which may lead to altered cell physiology and biochemistry, and extensive cellular DNA/ RNA and protein damage (Richa et al., 2016). The water-soluble transparent mycosporine-like amino acids (MAAs) are the most widespread intracellular UV-absorptive NPs synthesized by cyanobacterial species, exhibiting molecular weight ranging from 188 to 1050 Da (Singh et al., 2020; Pathak et al., 2019), while the hydrophobic scytonemins are less commonly found among cyanobacteria (Pathak et al., 2019). These UV-absorbing compounds harmlessly convert harmful solar energy into heat, avoiding the production of reactive oxygen species and thereby preventing oxidative processes linked to tumor formation (Bhatia et al., 2010). They also reduce the formation of UV-B-induced thymine dimer formation in hairless albino mice (Singh et al., 2020). Antioxidant properties (Rastogi et al., 2016) and antiproliferative effects on neoplastic cells (Singh et al., 2020) are some other characteristics exhibited by MAAs apart from UV screening activity. Scytonemin is a (naturally) dark brown UV-absorbing compound that appears in the outer mucilaginous sheath of cyanobacterial cells, particularly those bare to prolonged sunlight irradiation (Garcia-Pichel and Castenholz, 1991). Scytonemin not only provides UV protection but also delivers anti-inflammatory and anti-proliferative effects by inhibiting a serine/threonine kinase, specifically polo-like kinase-1, which is essential for mitotic spindle formation in fibroblasts (Stevenson et al., 2002). Detailed reports on photoprotective compounds derived from mangrove cyanobacteria are limited to the two aforementioned strains. As random screening is usually employed (methods in the quest for organisms producing bioactive compounds) is often incapable of removing recurring species in the initial stages (Bull et al., 1992), the polyphasic method of identification would probably help find new organisms producing such compounds.

### 4.5 Dyes and coloring agents

Biopigments and coloring agents derived from cyanobacteria, such as chlorophylls, carotenoids, and phycobiliproteins, have appeared as an alternative source of biodegradable colorants that are favorable for human health and environmentally sustainable, as opposed to chemically manufactured dyes, which are toxic to humans as well as the ecosystem (Saini et al., 2018; Mandal et al., 2020). Cyanobacterial chlorophylls (a, b, d, and f), on account of selectively absorbing light of the red and blue colors in the visible light spectrum, produce green and yellow pigments that are used as food color (Saini et al., 2018). They have also proved capable of functioning as a biomordant to increase the dyes of textiles, in addition to an antimicrobial colorant for fabrics (Miazek et al., 2015). Phycobiliproteins extracted from cyanobacteria are classified into different groups, such as allophycocyanin, allophycocyanin B, phycoerythrocyanin, blue C-phycocyanin, phycoerythrin, and phycoerythrin. All are water-soluble proteins that absorb red, orange, yellow, and green lights of the visible spectrum, respectively (Saini et al., 2018; Mandal et al., 2020). A phycocyanin extracted from *Oxynema aestuarii* obtained from the Indian Sundarbans was recently reported to show antioxidant activity (Hazra and Saha Kesh, 2017). This organism was identified using the polyphasic taxonomy approach. A mangrove-dwelling member of the genus *Acaryochloris* was also reported to contain chlorophyll-d as its major photosynthetic pigment (Larkum et al., 2012). Identification of this organism, however, is based solely on its 16S rRNA sequence.

### 4.6 Functional food

Driven by the urgent need for sustainable and scalable food sources in the wake of global population growth, there is increasing interest in exploring non-traditional alternatives beyond conventional crops. Growing concerns about feeding an expanding global population have intensified interest in functional foods as a promising way to meet nutritional needs sustainably, sparking increased research and consumer demand. This has promoted interest in the use of functional foods to meet the nutritional demands of the growing human population (Dixit and Suseela, 2013; Panjiar et al., 2017). Cyanobacteria (such as *Spirulina subsalsa*) and microalgae have long been consumed as functional foods due to their dense composition of essential nutrients, including polysaccharides, complete proteins, essential fatty acids, vitamins, and minerals supporting their role in human nutrition (Milledge, 2011). High levels of a multitude of nutrients, ease of cultivation without stringent requirements for freshwater or fertile farmland, and the ability of cyanobacteria and their products to withstand temperature and pH fluctuations make cyanobacteria an attractive candidate for exploration as a possible non-conventional source of nutrients (Panjiar et al., 2017). *Spirulina subsalsa*, a dominant cyanobacterial species found in three mangrove forests across the Southeastern coast of India, is identified as a source of carbohydrates, proteins, and lipids along with several important minerals and vitamins (Sakthivel and Kathiresan, 2013). However, these cyanobacteria were solely identified based on the taxonomic keys provided by Desikachary (1959). Polyphasic taxonomic studies of functional food-producing candidate cyanobacteria would be beneficial for accurate strain identification and assessing the non-toxic nature of cyanobacteria used in nutritional supplements by combining morphology, molecular phylogeny, chemistry, and ecology for preventing misidentification and detection of toxic or adulterant strains (Komárek, 2016). Brazilian mangrove forests harbor a member of the genus *Synechocystis*, a known producer of significant amounts of fatty acids such as linoleic acid and alpha-linolenic acid that have gained importance as nutritional supplements (Matsudo et al., 2020). A member of the genus *Cyanobium* (occurring in Brazilian mangroves) is a source of adipic acid, which is utilized in the bakery industry as a leavening compound and an acidulant (Armstrong et al., 2019).

### 4.7 Biofuels

Fossil fuel usage has been proven to cause several large-scale global disturbances, namely a rise in the average global temperature, alterations in climate patterns, and adverse impacts on the environment caused by mining operations to recover coal and petroleum for use (Behera et al., 2015; Martens, 2013; Sitther et al., 2020). Thus, the quest for alternative, clean, and renewable sources of energy has prompted the exploration of other directly biologically derived options, such as fats derived from food crops, viz., soybean oil, sugarcane lipids, and corn oils, which are subsequently used as feedstocks for producing diesel alternatives and ethanol (Sitther et al., 2020; Goldemberg, 2007). The idea of cyanobacterial cells used as a potential biofuel source is increasingly gaining popularity, as they might prove to have shorter generation time than common food crops, they are capable of growing in minimal light and nutrient availability, and do not compete for agricultural fields with other valuable cash crops (Sitther et al., 2020; Rodolfi et al., 2009). The biodiesel extracted from cyanobacteria has also proved to be eco-friendly as it has significantly lower levels of sulfur and aromatic hydrocarbon emissions, and efficient combustion (Sitther et al., 2020). Various methods of biochemical processing can be used on the cyanobacterial biomass in order to manufacture a broad range of energy sources such as biogas, biohydrogen, cellulosic ethanol, and biodiesel (Machado and Atsumi, 2012). Cyanobacterial isolates from the sugars can readily ferment into bioethanol, while cellular lipids can be chemically transformed into fatty acid methyl esters (FAMEs), which are the primary constituents of biodiesel. The above-mentioned advantages coupled with the efficacy of capture and transformation of solar energy to fuels make cyanobacteria the candidates of choice for exploitation, offering significant financial and logistical benefits compared to the manufacture of biofuel from cash crops (Sitther et al., 2020; de Farias Silva and Bertucco, 2016). Mangrove-dwelling members of the genera *Oxynema, Nostoc, Synechocystis,* and *Cyanobium* have recently been reported to produce intermediates important in biofuel production (Matsudo et al., 2020; Armstrong et al., 2019). Cyanobacterial polyphasic taxonomy in combination with phylogenomics helped in the precise identification of conserved functional genes responsible for the production of polyunsaturated fatty acids (PUFAs) in the diatom *Phaeodactylum tricornutum*, and linked these genes to well‑characterized taxa (Rehmanji et al., 2022). This integrated approach is anticipated to facilitate the understanding of mangrove cyanobacterial metabolism and guide targeted enhancements to boost yields in cyanobacterial biorefineries (Zammit et al., 2023).

### 4.8 Bio-fertilizers

Sustainable agriculture is currently an urgent need as the earth strains to recover from the damage caused by claiming more land for agricultural purposes and enhancing the productivity of land already under cultivation using synthetic fertilizers and pesticides. Food security is essential for the purpose of accommodating and feeding the staggering global population of ∼7.9 billion people (Joshi et al., 2020). Bio-fertilizers are gaining popularity as sustainable, eco-friendly fertilizers (as opposed to chemical fertilizers) as they can produce a host of beneficial organic compounds in soil that stimulate the growth of seeds and plants and also maintain a proper balance of macro-nutrients nitrogen, phosphate, potassium (NPK), and other micronutrients (Joshi et al., 2020). Mangrove cyanobacteria have added advantages, such as secretion of plant hormone like Indole-3-acetic acid (IAA) from *Phormidium* sp. (identified by 16S rRNA analysis) (Boopathi et al., 2013), and other secondary metabolites (such as seed and foliage lipid content enhancer secreted by *Microcoleus chthonoplastes* isolated from a Mexican mangrove forest) (Bashan et al., 2000) that supplement healthy crop growth; fixing of atmospheric nitrogen in the soil (notably by *Microcoleus chthonoplastes*) to replenish the nitrogen content of agricultural fields (Bashan et al., 1998; Kathiresan, 2000); and acting as a biocontrol agent against phytopathogens by secreting antifungal and antibacterial NPs (Sakthivel and Kathiresan, 2015; Joshi et al., 2020). Cyanobacteria may also act as soil stabilizers by forming three-dimensional extracellular polysaccharide (EPS) filamentous sheaths (Lynch and Bragg, 1985). Cyanobacteria used in agriculture as cost-effective alternatives to harmful chemically synthesized fertilizers benefit the environment, as well as human and livestock health by recycling nutrients and restoring soil fertility through the benign method of nitrogen fixation, heading towards the improved production of nutritious and safe-to-consume food (Joshi et al., 2020; Bashan et al., 1998). Additional systematic and phylogenomic investigation of mangrove-dwelling members of *Phormidium, Microcoleus,* and *Oscillatoria* genera may prove profitable for agricultural applications, as well as for determining the roles of these cyanobacteria in the mangrove ecosystem (Joshi et al., 2020). It is also expected to be helpful to identify lineages with metabolic pathways relevant to IAA and EPS production and mapping these genes across clades of neighbouring cyanobacterial strains, thus broadening the candidate pool for biofertilizer production (Zammit et al., 2023).

### 4.9 Polyhydroxyalkanoate bioplastics

Plastics are used in most aspects of daily life, owing to their versatile mechanical properties, durability, thermal and electrical insulating properties, lightweight, and cheap production costs (Samantaray et al., 2014). However, wastes generated from plastic articles do not easily undergo natural microbe-mediated decomposition (i.e., biodegradation) (Barnes et al., 2009). Attempts to dispose of petrochemical-derived plastics by incineration lead to the production of toxic gases such as dioxins, polycyclic aromatic hydrocarbons (PAHs), furans, and hydrogen cyanide ((Samantaray et al., 2014; Barnes et al., 2009). The increasing awareness about the detrimental effects of the petroleum-based plastic wastes on the environment has led to the search for novel raw materials that can be utilized for the manufacture of plastics developed from biodegradable polymers (Lee, 1996, Samantaray et al., 2014). Polyhydroxyalkanoates (PHAs) are polyesters of hydroxyl acids produced and accumulated intracellularly by several prokaryotes (including cyanobacteria) and are completely degraded by environmental microbiota (Lee, 1996). The products made out of PHAs are suitable for numerous applications, including the production of medical implants, drug delivery carriers, printing, and photographic material, as well as more conventional practices in the production of packing and single-use materials (Samantaray et al., 2014; Wang et al., 2008; Yao et al., 2008). Members of *Synechocystis* isolated from a Brazilian mangrove environment have been shown to produce and accumulate high volumes of PHAs when subjected to high-intensity illumination (Gracioso et al., 2021). Further improvement of these strains for scaling up bioplastic production, field application, and realistic performance predictions requires meticulous exploration of their physiological and ecological parameters and tolerance levels, which is an integral part of polyphasic taxonomy (Komárek, 2016).

### 4.10 Environmental detoxification

Cyanobacteria are gaining popularity as an effective tool for the elimination of cytotoxic heavy metals coming from the groundwater, which are discharged mainly as a result of improper industrial effluent disposal containing huge volumes of toxic heavy metals eg, mangrove-derived *Synechococcus elongatus* and *Gloeocapsa* sp. effectively remove lead and cadmium, respectively (Genuário et al., 2019; Anburaj et al., 2020, 2017). All the strains were identified based on their external morphology. Heavy metals cause various disorders in both humans and wildlife, and several species of heavy metals are capable of causing a thinning of eggshells (Faber and Hickey, 1973). Cyanobacteria-mediated bioremediation involves highly efficient uptake of complex pollutants using sensitive polysaccharide receptors on their surface (Malik, 2004). The trapped pollutants are then bound passively to the cellular structure of the cyanobacterial cell aggregates through biosorption (Malik, 2004; Kulal et al., 2020). Mangrove-dwelling cyanobacteria are also garnering attention as potential degraders of some dyes, eg, a Brazilian species of the *Phormidium* genus has been reported to effectively decolorize indigo blue (Silva-Stenico et al., 2012). The current identification of these strains, however, was based on utilizing the 16S rRNA sequence only, highlighting a limited understanding of the taxonomical position of these cyanobacteria. Polyphasic taxonomy is anticipated to be helpful in correctly linking pollutant-degrading phenotypes to relevant cyanobacterial strains for reproducible bioremediation studies and further upscaling (Komárek, 2016; Zammit et al., 2023). A species of *Phormidium* derived from an Indian mangrove forest has also been reported to effectively degrade the textile dye malachite green (Kulal et al., 2020).

## 5 Conclusion: challenges and future scope

Cyanobacteria represent an ancient lineage of a morphologically varied group of photosynthetic, Gram-negative bacteria that have functioned as key players in the evolution of life forms on Earth through oxygenic photosynthesis, and they still play a role of paramount importance in the universal carbon and nitrogen cycles (Ducat et al., 2011). Approximately 50% of the ocean’s primary productivity is supplied by cyanobacteria, and they are potential alternative sources of various commercially and pharmaceutically important NPs (Pramanik et al., 2011; Jones et al., 2021). Despite having a ubiquitous presence, Cyanobacterial biotechnology and natural product (NP) discovery face technical and practical barriers that limit both NP discovery and commercial translation. Only a small subset of cyanobacteria is suitable for genetic engineering, limiting activation of BGCs and the scope of strain improvement, while many desirable metabolites appear at low, inconsistent titers that complicate purification and scale‑up (Covington et al., 2021; Romano et al., 2018). Heterologous expression is often solely experimental and resource‑intensive, requiring host redesign for compatible promoters, activating enzymes, and precursor supply, and bypassing cloning and expression challenges for large NRPS/PKS clusters (Liu and Pakrasi, 2018; Wells et al., 2018; Yang et al., 2017). Large-scale cultivation poses further obstacles: high water, nutrient, and energy inputs, contamination and phage risk, and ecological stability concerns in open systems, which undermine reliability (Iwaki et al., 2006; Qiang et al., 1998; Zahra et al., 2020). Miniaturized OSMAC screens, microfluidics, in vitro reconstitution and chemo‑enzymatic synthesis can circumvent some culture limitations and accelerate mechanistic insight, but these approaches may be costly, hard to scale and carry environmental trade‑offs from chemical steps (Baunach et al., 2024). Integrated strategies involving multiomics, enzyme engineering, and scalable, low‑impact workflows are therefore essential to bridge discovery with sustainable industrial use (Zahra et al., 2020).

## Funding

This work was supported by the Council of Scientific and Industrial Research (CSIR), India, in the form of NET-Senior Research fellowship to ARR [09/096(0997)/2019-EMR-I] and the University Grants Commission (UGC), India, in the form of NET-Senior Research fellowship to SB [award number 200510616803].

## CRediT authorship contribution statement

**Arup Ratan Roy:** Data curation, Methodology, Writing – original draft. **Shayontani Basu:** Data curation, Methodology, Writing – original draft. **Sergio de los Santos Villalobos:** Writing – review & editing. **Joydeep Mukherjee:** Supervision, Writing – review & editing.

## Declaration of competing interest

The authors declare that they have no known competing financial interests or personal relationships that could have appeared to influence the work reported in this paper.
